# Differential impacts of regenerative agriculture practices on soil organic carbon: a meta-analysis of studies from India

**DOI:** 10.1038/s41598-025-12149-6

**Published:** 2025-09-29

**Authors:** Mukund Patil, Cuba Perumal, Pushpajeet Choudhari, Rajesh Pasumarthi, Gajanan Sawargaonkar, Ramesh Singh

**Affiliations:** International Crops Research Institute for the Semi-Arid Tropics, Patancheru, India

**Keywords:** Regenerative agriculture, Meta-analysis, Crop residue, FYM, Compost, Tillage, Carbon sequestration, Environmental impact, Climate-change mitigation, Sustainability

## Abstract

Regenerative agriculture (RA) is heralded as a transformative solution to combat climate change, enhance biodiversity, and improve soil health. However, its effectiveness across diverse agro-climatic contexts remains underexplored. This meta-analysis synthesizes results from 147 peer-reviewed studies across India’s major agro-ecological and agro-climatic regions. Using a random-effects model, we estimate the soil organic carbon (SOC) change attributable to a suite of RA practices, including organic amendments (farmyard manure, green manure, compost, and biochar), conservation tillage, crop residue retention, and fertilizer management. Biochar application resulted in the highest SOC gain, followed by farmyard manure, green manure, compost, and fertilizer management. Conservation tillage and crop residue retention demonstrated moderate, yet consistent, carbon benefits across time scales. The SOC gains were most significant over durations exceeding five years and varied across agro-ecological regions, with semi-arid and sub-humid regions showing particularly strong responses. The findings affirm that RA practices effectively sequester carbon, particularly when applied over longer durations and in regionally adapted combinations.

## Introduction

Soil carbon sequestration is now widely recognized as a cornerstone of nature-based climate solutions. Enhancing soil organic carbon (SOC) not only offsets a portion of anthropogenic ${\textrm{CO}}_{2}$ emissions but also amplifies soil fertility, water retention, and yield stability. As agriculture accounts for approximately 24% of global emissions when including land-use change, transitioning to carbon-sequestering farming systems is an essential component of global mitigation strategies. The “4 per 1000” initiative and IPCC reports have emphasized the need for actionable soil-centric approaches to achieve near- and long-term climate goals^1^. Among these approaches, regenerative agriculture (RA) as a nature-based solution that restores soil health while improving productivity has gained particular momentum.

Regenerative agriculture has garnered increasing global interest as a sustainable farming paradigm that not only sustains productivity but also actively restores soil health, biodiversity, and ecosystem functions^2^. Defined by practices such as cover cropping, minimal tillage, diversified crop rotations, soil organic amendments, and agroforestry, RA offers a holistic approach to mitigate climate change by sequestering atmospheric carbon dioxide and reducing greenhouse gas (GHG) emissions from agricultural systems^3–6^. These practices contribute to long-term SOC accumulation, improved soil structure and moisture retention, and enhanced agroecosystem resilience. Beyond ecological benefits, RA is increasingly linked to economic incentives through its integration with carbon markets^7,8^. Carbon farming initiatives, which reward farmers for increasing SOC or reducing emissions, are emerging globally as a means to align sustainability with profitability^9^.

Several individual studies have demonstrated the benefits of RA practices for enhancing SOC, but the magnitude, consistency, and context-dependency of these outcomes remain poorly understood, especially across India’s diverse agro-ecological and agro-climatic regions^10^. The carbon sequestration potential of RA practices is highly context-specific, influenced by factors such as soil type, cropping system, climate, and duration of practice. For instance, SOC responses may vary significantly between intensively cultivated rice–wheat systems of the Indo-Gangetic Plains, the rainfed cropping systems of central India, and the arid soils of Rajasthan, where limited moisture and high temperatures accelerate organic matter decomposition. This variability is particularly pronounced in tropical and sub-tropical regions, including India, where high decomposition rates and diverse land use patterns can affect carbon dynamics. While several meta-analyses have synthesized the effects of conservation and regenerative practices on SOC, they have predominantly relied on data from temperate regions or globally aggregated datasets^10–15^. Meta-analyses that focus exclusively on data from India are rare, resulting in a critical knowledge gap in understanding region-specific outcomes. This lack of localized synthesis constrains the development of evidence-based agricultural and climate mitigation policies tailored to the unique conditions of Indian agriculture.

To address this evidence gap, authors conducted a comprehensive meta-analysis of 147 peer-reviewed articles with field studies spanning varied agro-ecosystems across India to evaluate the efficacy of key RA practices in enhancing SOC sequestration. Authors have quantified effect sizes across different practices, durations, and climatic regions and assessed synergistic impacts of bundled interventions. This analysis highlights spatial and temporal patterns in SOC responses, offering critical insights for designing context-appropriate RA strategies. By synthesizing a robust evidence base, this study aims to inform scalable, regionally tailored interventions that support India’s soil health restoration and climate mitigation goals through nature-based solutions. Specifically, the objective of present study is to quantify SOC outcomes of RA practices across India’s agro-ecological zones and identify the most context-effective strategies. Addressing dimensions that have not been thoroughly explored in prior syntheses, this study provides novelty through its exclusive focus on Indian agro-ecosystems, use of a comprehensive India-specific dataset, and systematic evaluation of both individual and bundled regenerative practices.

## Results

### Dataset description

**Fig. 1:**
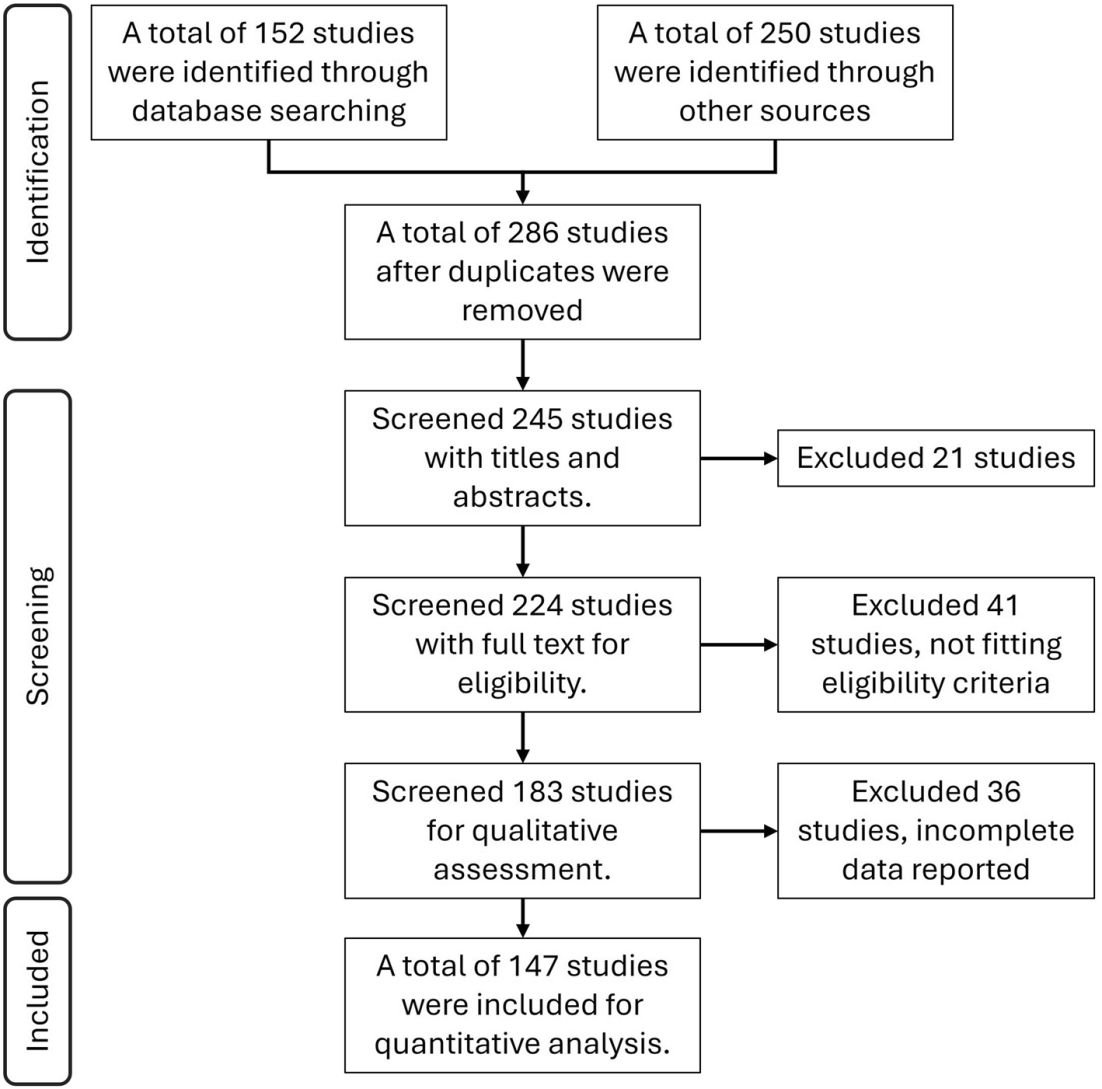
PRISMA 2020 flow diagram illustrating the systematic process of study identification, screening, and inclusion for the meta-analysis.

Figure 1 illustrates the systematic process of study identification, screening, and inclusion for the meta-analysis as per the PRISMA 2020 statement. A total of 402 articles were screened for carbon sequestration, out of which 224 articles were found to be relevant for the Indian scenario. The final selection of studies was done based on pre-defined inclusion and exclusion criteria. Finally, 147 studies were selected for quantitative analysis with 1020 data pairs to evaluate the effects of RA practices on SOC across India (Fig. 1). The compiled dataset draws from a wide range of agro-ecological and agro-climatic regions, capturing the country’s considerable environmental and agronomic diversity. Studies were selected through a rigorous screening process to ensure methodological transparency, completeness of data, and broad geographic representation. For each study, detailed metadata were extracted, including bibliographic information, geographic coordinates, and study design characteristics. Particular attention was given to documenting RA practice descriptors, such as farmyard manure (FYM), green manure, compost, biochar, minimum tillage, zero tillage, crop residue retention, and fertilizer management. The summary of study characteristics included in the meta-analysis is presented in Table 1. Manure-based amendments, including FYM, green manure, and compost, were the most frequently assessed practices (61%), followed by fertilizer management interventions (50%), crop residue retention (41%), and reduced or zero tillage (27%). Biochar-related studies were relatively few, comprising only 7% of the dataset (Table 1).

**Table 1:** Summary of study characteristics included in the meta-analysis (n = 147).

Category	Description	Number of studies	% of total
Agro-climatic regions (ACRs)	Top five regions based on number of studies: ACR-6 Trans Gangetic plain; ACR-4 Middle Gangetic Plain; ACR-2 Eastern Himalaya; ACR-9 Western Plateau and hills; ACR-10 Southern Plateau and Hills	97	65%
Agro-ecological regions (AERs)	Top five regions based on number of studies: AER-4 Northern plain and central highlands; AER-6 Deccan Plateau (hot semi-arid); AER-15 Assam and Bengal Plain (hot sub-humid); AER-14 Western Himalaya (warm sub-humid); AER-9 Northern Plain (hot sub-humid)	97	65%
RA practice type	Manure (FYM, green manure, compost)	90	61%
	Biochar (pyrolyzed biomass)	10	7%
	Tillage (zero or minimum tillage)	40	27%
	Crop residue retention	60	41%
	Fertilizer management	74	50%
Cropping systems	Cereal–cereal	71	48%
	Cereal–legume	39	27%
	Cereals only	15	10%
	Other (legumes/oilseeds/vegetables)	22	15%
SOC baseline range	Baseline soil organic carbon (% or g/kg)	0.02%–2.1%	—
Study duration	$\le$ 5 years (short-term studies)	51	35%
	6–10 years (medium-term studies)	37	25%
	$>10$ years (long-term studies)	22	15%

Additional variables such as study duration, agro-ecological and agro-climatic classifications of study location, control treatment descriptions, and cropping systems were recorded to support subgroup analyses and address potential sources of heterogeneity. Notably, 65% of the studies were clustered within five key agro-climatic regions: the Trans-Gangetic Plain (ACR-6), Middle Gangetic Plain (ACR-4), Eastern Himalaya (ACR-2), Western Plateau and Hills (ACR-9), and Southern Plateau and Hills (ACR-10). These largely align with major agro-ecological regions such as AER-4 (Northern Plain and Central Highlands), AER-6 (Deccan Plateau, hot semi-arid), AER-15 (Assam and Bengal Plain, hot sub-humid), AER-14 (Western Himalaya, warm sub-humid), and AER-9 (Northern Plain, hot sub-humid). The majority of studies focused on cereal-based cropping systems, particularly cereal–cereal (48%) and cereal–legume (27%) rotations. SOC was the primary outcome variable, with extracted data including mean and standard deviation values for both treatment and control groups. Baseline SOC levels varied widely, ranging from 0.02 to 2.1%, reflecting the diversity in initial soil conditions. In terms of temporal coverage, short-term studies ($\le$5 years) comprised 35% of the dataset, while medium-term (6–10 years) and long-term ($>10$ years) studies accounted for 25% and 40%, respectively.

### Meta-analysis outcomes

**Fig. 2:**
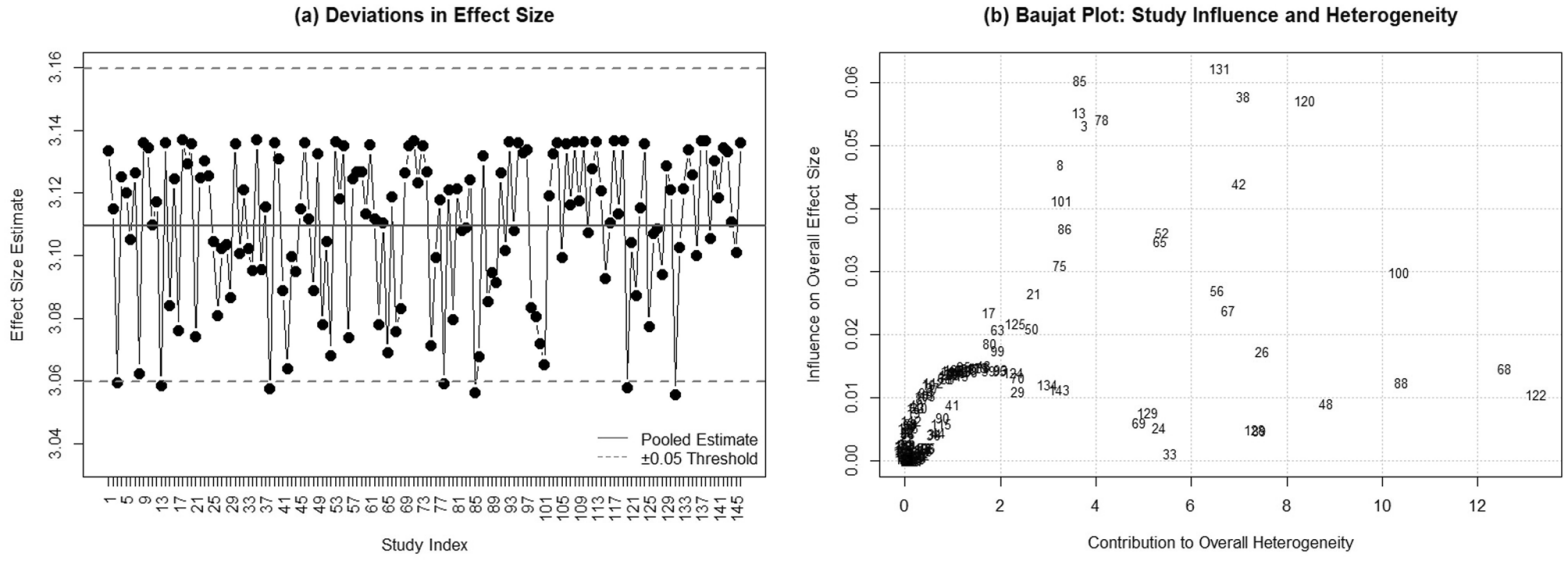
Results from sensitivity analysis: (**a**) effect size outcomes from leaving out a study while meta-analysis, and (**b**) Baujat plot.

The results from a comprehensive sensitivity analysis conducted using Leave-One-Out (LOO) diagnostics and the Baujat plot are presented in Fig. 2a, b, respectively. The LOO analysis revealed that a few studies exhibited a deviation in the overall effect size, up to -0.05, indicating a moderately influential effect when excluded from the model, while no study showed positive deviations exceeding +0.05 (Fig.2a). The Baujat plot (Fig.2b) indicated four studies appeared in the top-right quadrant, with a heterogeneity contribution exceeding 6 and an influence on the overall effect size greater than 0.04. Finally, the outcome from ‘influence ()’ function indicated that none of the studies had influenced the overall effect size. Based on convergence across these diagnostics, the small deviations in effect size observed in LOO analysis for a few studies were ignored, and all the studies were retained for further analysis.

Multiple meta-analytic models were tested using distinct methods for between-study variance estimation. Performance matrix for different methods of estimating variance is presented in Table 2. The REML method produced an effect size estimate of 3.11 with a standard error (SE) of 0.22 and moderate heterogeneity ($I^{2} = 75.6\%, \tau ^{2} = 4.58$). This was closely aligned with the ML and DL methods, both of which estimated similar pooled effects (3.07–3.10) and comparable heterogeneity levels ($I^{2} \sim 73--75\%$). The HS and HSk also provided consistent estimates, suggesting these methods perform similarly under the current data structure.

In contrast, the SJ and HE estimators generated considerably higher effect size estimates (4.26 and 4.62, respectively), with substantially greater heterogeneity ($I^{2}> 96\%, \tau ^{2} > 38$). These inflated values may reflect the known tendency of SJ and HE methods to overestimate heterogeneity, especially in datasets with high variability or small sample sizes^16^. The EB, PM, and PMM approaches yielded intermediate estimates ($\sim$3.8) with high heterogeneity ($I^{2} \sim 93\%$), suggesting a more conservative estimation of effect sizes under greater uncertainty. Given its balance between precision and heterogeneity, the REML model was deemed most appropriate for this study. It avoids underestimation of between-study variance (as seen with DL) and overestimation (as with SJ and HE), providing a stable and widely recommended choice for complex meta-analyses^17^.

Application of the trim-and-fill method to adjust for potential publication bias resulted in a slight reduction in estimated effect sizes across most sub-groups. For instance, the overall effect estimate decreased from 3.46 to 1.98 after filling 296 data points on the negative side of outcomes. In practice-specific studies, the effect dropped from 4.21 to 3.73 after correcting for 3 potentially missing data points. More substantial corrections were also observed in two-level groupings, such as AER + RA practice (34 imputed points) and ACR + RA practice (30 imputed points), highlighting the presence of potential reporting bias^18^. Nonetheless, even after these corrections, all effect sizes remained significant and positive, indicating the robustness of RA benefits. These results further affirm that RA practices offer reliable enhancements in SOC across different regions and conditions.

**Table 2:** Performance matrix for different methods of estimating variance.

Method	Estimate	SE	CI.lb	CI.ub	$\tau ^2$	$I^2$ (%)
REML	3.11	0.22	2.68	3.54	4.58	75.6
DL	3.07	0.21	2.65	3.48	4.17	73.9
ML	3.10	0.22	2.67	3.53	4.49	75.3
SJ	4.26	0.55	3.20	5.33	38.58	96.3
HS	3.06	0.21	2.64	3.48	4.11	73.6
HE	4.62	0.72	3.20	6.03	70.23	97.9
HSk	3.06	0.21	2.65	3.48	4.15	73.8
EB	3.85	0.40	3.08	4.63	18.98	92.8
PM	3.85	0.40	3.08	4.63	18.98	92.8
PMM	3.86	0.40	3.08	4.64	19.18	92.9

*REML* restricted maximum likelihood, *ML* maximum likelihood, *DL* DerSimonian–Laird, *SJ* Sidik–Jonkman, *HS* Hunter–Schmidt, *HE* Hedges, *HSk* Hunter–Schmidt (corrected), *EB* empirical Bayes, *PM* Paule–Mandel, *PMM* median-unbiased Paule-Mandel.

### Effectiveness of RA practices

The results of the meta-analyses across different sub-groups are presented in Table 3, with values in parentheses indicating outcomes adjusted using the trim-and-fill method. The pooled estimate across all studies (k = 1020) suggests a strong positive effect of RA practices on soil carbon sequestration, with a mean effect size of 3.46 (standard error [SE] = 0.10, 95% confidence interval [CI]: 3.25–3.66). Considering a mean pooled standard deviation of 0.049, this effect size translates to an approximate 0.17 percentage point increase in SOC, representing a 17% gain over the baseline SOC level in control conditions. However, the analysis indicates substantial heterogeneity ($I^2$ = 86.4%), reflecting possible influences of climatic conditions, soil types, duration for which practice is adopted, and specific RA practices. The sub-group analysis based on the duration of studies, individual RA practices, agro-ecological regions (AERs), and agro-climatic regions (ACRs) revealed the reduction in heterogeneity. Among these analyses, the effect size for the study duration sub-group was highest (Estimate = 5.21, CI = 3.07 - 7.35, $I^2$ = 29.5%), indicating that time is a key factor for enhancing the effectiveness of RA practices.

**Fig. 3:**
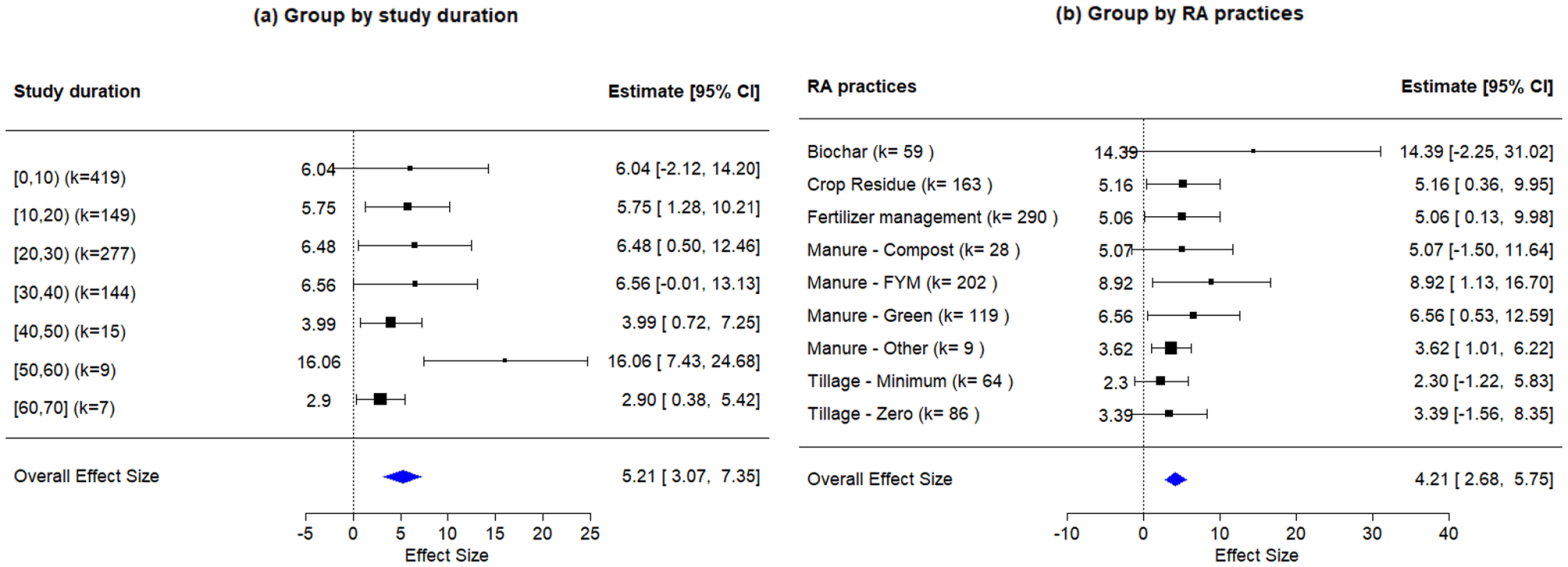
Forest plots of the meta-analysis for data grouped by: (**a**) study duration and (**b**) regenerative agricultural practices.

Figure 3-a presents the forest plot of the meta-analysis for data grouped by study duration. The effect sizes were higher in studies with long-term experiments spanning 30 to 40 years (Estimate = 6.56, k = 144, CI= -0.01–13.13) and 20–30 years (estimate = 6.48, k = 277, CI = 0.50–12.46), while initial ($<10$ years) implementation also yielded considerable gains (Fig. 3a). These results align with the inherently nonlinear trajectory of soil carbon accumulation, characterized by accelerated gains in the early years followed by a gradual stabilization as soils approach a saturation point or equilibrium^19^. Interestingly, the 50–60 year category reported the highest effect estimate (estimate=16.1), though it had high variance and a small number of studies (k = 9, CI = 7.43–24.68). The decline in the 60–70 year group (estimate = 2.90, k = 7, CI = 0.38–5.42) might indicate SOC stabilization or data sparsity rather than actual reduction. These findings are particularly relevant for smallholder farmers, who often face financial limitations and prioritize short-term returns. The evidence supporting the medium-term (20–40 years) effectiveness of RA can inform policies that provide transitional support and incentives tailored to smallholder needs. Whereas, long-term commitment to RA practices can enhance soil health, productivity, and climate resilience in resource-constrained, rainfed, and semi-arid farming systems.

### Effectiveness of specific RA practices

The sub-group analysis by RA practices revealed that the results reported across the studies are similar, as the variability was reduced to zero (Table 3). Among the various practices assessed, biochar application exhibited the highest effect size (estimate = 14.4; k = 59, CI = $-2.25$ to 31.02), indicating its strong potential to enhance SOC through its inherent carbon-rich composition and long-term stability in soils. This aligns with previous findings that suggested biochar’s long-term carbon stability and its role in improving soil physical and chemical properties^20–22^. However, this notably high effect size is largely derived from short-term studies. Practical challenges such as the high cost of biochar production, variability in biochar feedstock and application methods, and limited accessibility for smallholder farmers may constrain its widespread adoption for SOC improvement. Additionally, potential confounding factors such as publication bias and site-specific responses further emphasize the need for careful consideration.

Figure 3b presents the forest plot of the meta-analysis for data grouped by RA practices. Manure-based amendments, particularly FYM (estimate = 8.92; k = 202, CI = 1.13–16.7) and green manure (estimate = 6.56; k = 119, CI = 0.53–12.59), also demonstrated substantial SOC gains, reinforcing the evidence that organic matter inputs are crucial for building soil carbon stocks^23^. Compost-based manure showed a moderate impact (estimate = 5.07, k = 28, CI = $-1.5$ to 11.64), likely reflecting variability in quantity of compost applied, compost maturity, composition, and application methods^24,25^. Fertilizer management (estimate = 5.06, k = 289, CI = 0.13–9.98) and crop residue retention (estimate = 5.15, k = 164, CI = 0.36–9.95) also reported consistent and positive effects, suggesting that integrated nutrient management, balancing organic and inorganic sources, can effectively promote SOC accumulation^26–36^. On the other hand, tillage-based interventions showed relatively lower impact. The zero or minimum tillage is often promoted for soil and moisture conservation, but its standalone effect on SOC buildup may be limited, especially in coarse-textured or low-residue systems^37–40^. However, when combined with residue retention or cover cropping, tillage reduction can yield synergistic benefits in SOC improvements^41^.

**Fig. 4:**
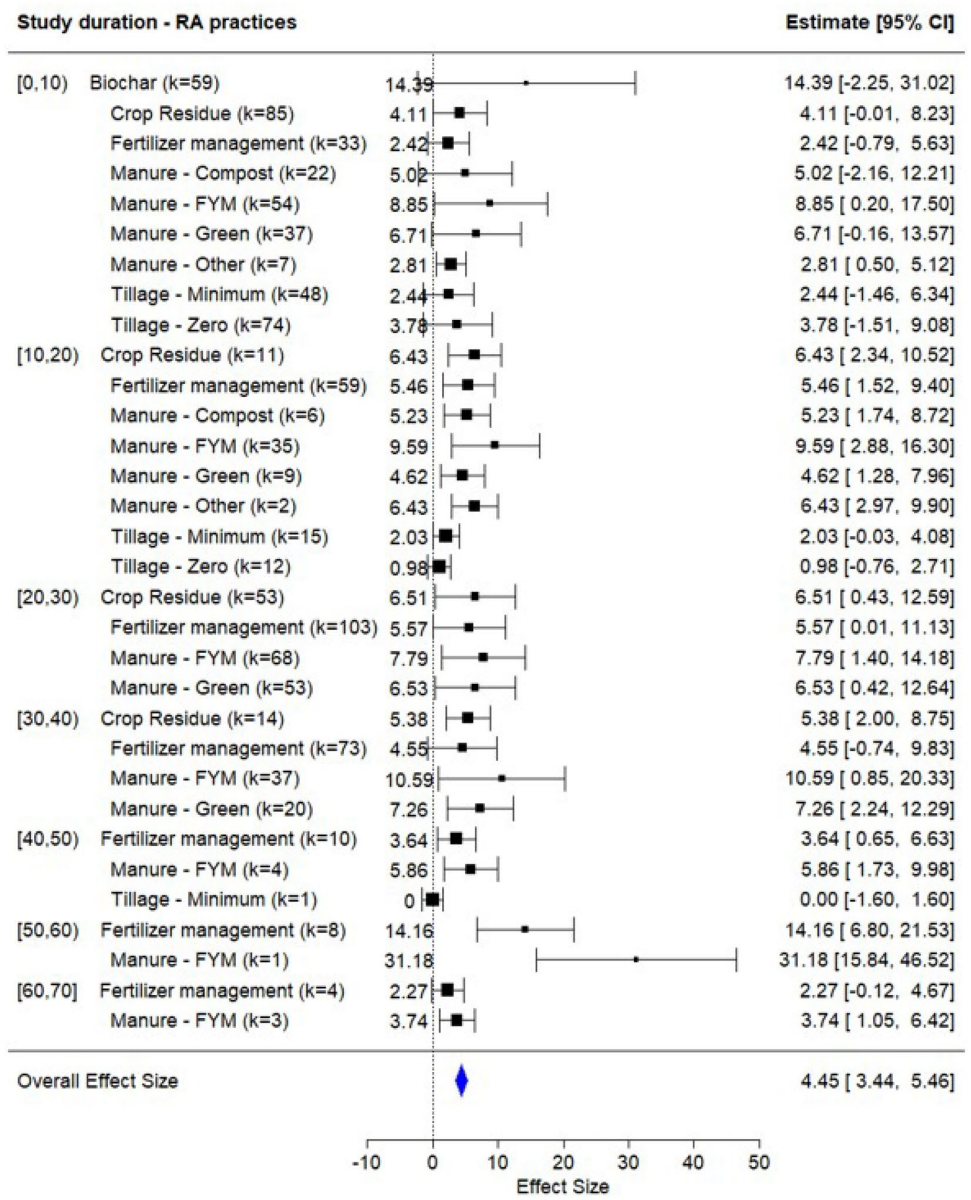
Forest plot of meta-analysis result for data grouped by study duration and regenerative agricultural practices.

The effectiveness of these RA practices was further varied by the duration over which they were implemented, as illustrated in Fig. 4. In the short-term (0–10 years), biochar application demonstrated the highest SOC gain (estimate = 14.4, k = 59, CI = $-2.25$ to 31.02), albeit with high variability (72%), suggesting that its effect is strongly dependent on contextual factors such as biochar type and soil conditions^21,42,43^. Among organic amendments, FYM showed a consistently high effect size (estimate = 8.85, k = 54, CI = 0.2–17.5), followed by green manure (estimate = 6.71, k = 37, CI = $-0.16$ to 13.57) and compost (estimate = 5.02, k = 22, CI = $-2.16$ to 12.21), indicating their rapid impact on SOC levels even within a decade of application^44–46^. Tillage reduction practices exhibited modest short-term benefits^47,48^, likely due to the time required for soil structural changes and microbial communities to stabilize^49,50^. Crop residue management also yielded moderate short-term gains (estimate = 4.11, k = 85, CI = $-0.01$ to 8.23), as the processes of crop residue decomposition into soil organic matter are generally slower.

As study durations extended into the medium term (10–30 years), the SOC benefits of RA practices became more pronounced and stable. FYM maintained strong performance (estimate = 9.59 at 10 to 20 years and 7.79 at 20 to 30 years), suggesting cumulative benefits from repeated applications and organic matter build-up^44^. Crop residue and fertilizer management practices also showed improved effect sizes during this period (estimate = 6.43–6.51 and 5.46–5.57, respectively), likely due to enhanced nutrient cycling and increased microbial biomass over time^51–54^. Interestingly, the effectiveness of zero tillage declined at 10–20 years (Estimate = 0.98, k = 12, CI = $-0.76$ to 2.71), indicating that minimal tillage alone may not sustain SOC improvements unless combined with organic matter inputs^39,55^. Often, zero-tillage practices are periodically interrupted by conventional tillage for agronomic purposes such as weed control or soil compaction management, which can negate previously accrued soil carbon benefits^56^.

In long-term studies (30–60+ years), the most substantial SOC gains were observed, particularly for FYM, which peaked at an effect size of 31.2 at 50–60 years, although this estimate was based on a single study^57^. Fertilizer management also demonstrated substantial long-term effects (14.2), suggesting that balanced nutrient application may enhance SOC accumulation through improved crop productivity and nutrient cycling^15,45^. Green manure continued to provide consistent benefits across time scales, peaking at 7.26 in the 30–40 year range. In contrast, the performance of tillage-based interventions diminished over time, reinforcing the notion that tillage reduction must be integrated with organic inputs to deliver sustained SOC gains.

**Fig. 5:**
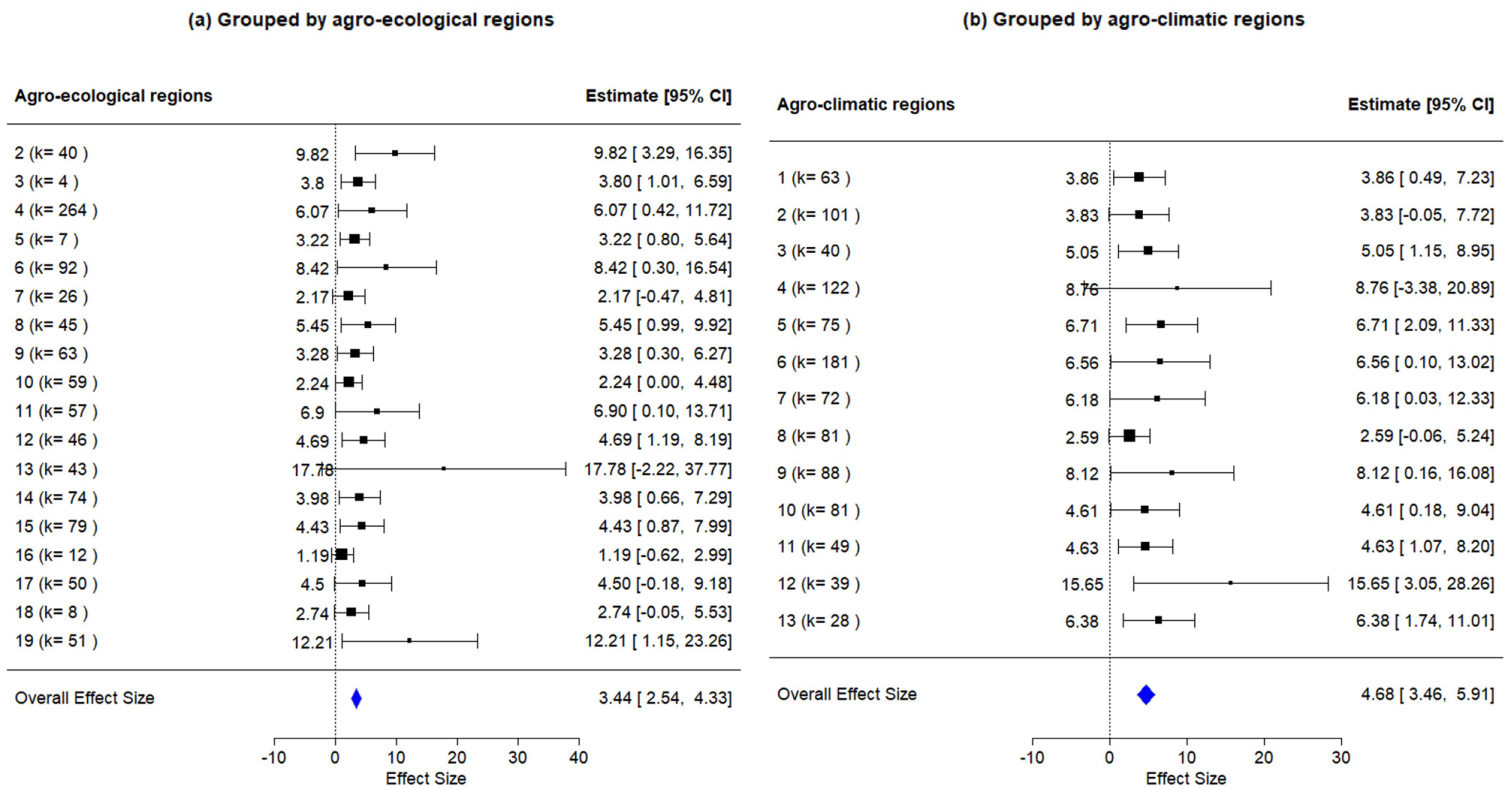
Forest plot of meta-analysis result for data grouped by: (**a**) agro-ecological regions and (**b**) agro-climatic regions.

### Effectiveness of RA practices across agro-ecological and agro-climatic regions

**Fig. 6:**
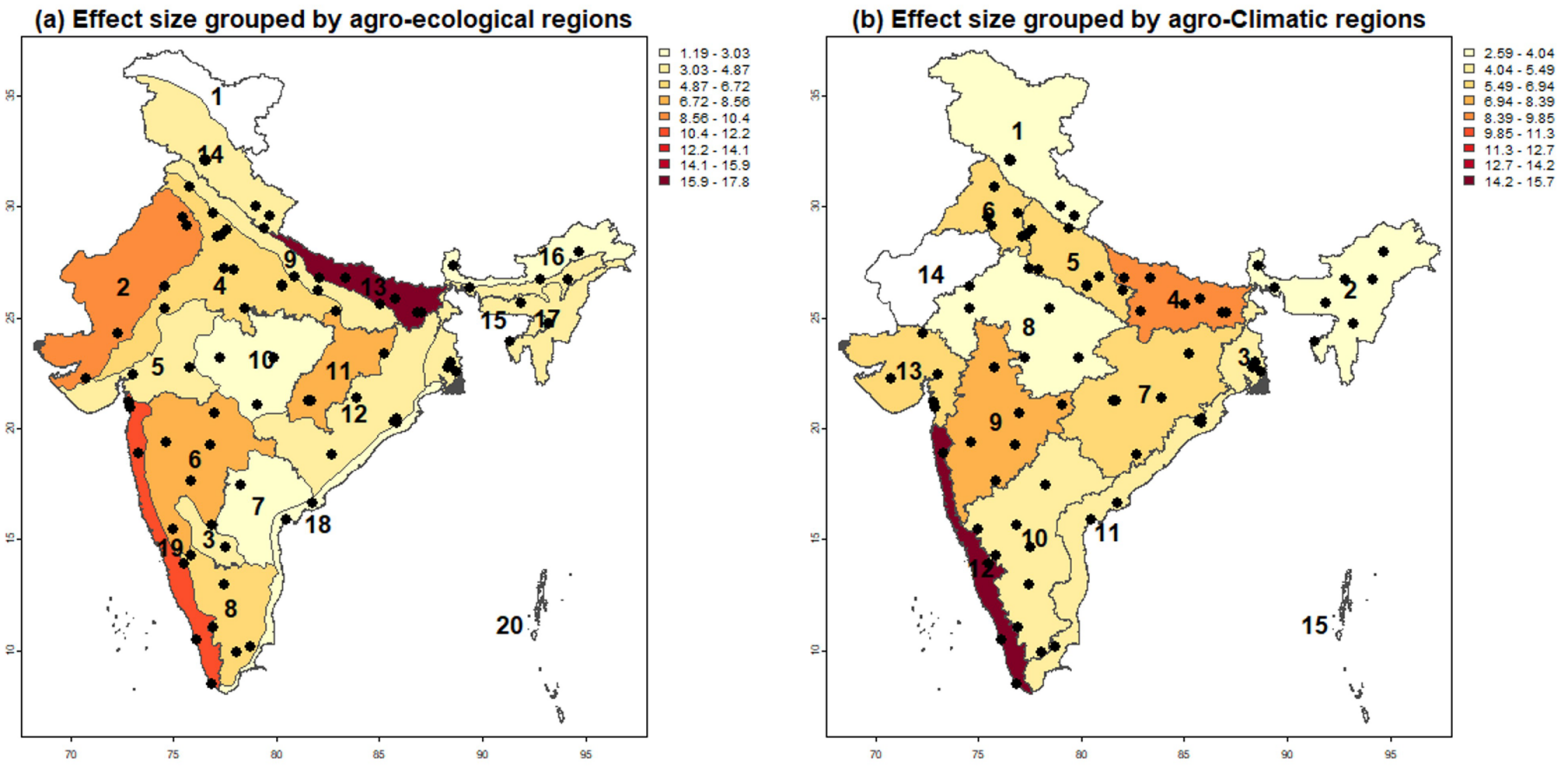
Spatial representation of average effect sizes grouped by: (**a**) agro-ecological regions and (**b**) agro-climatic regions (source for regions’ boundaries: https://www.data.gov.in).

The results from meta-analysis for data grouped by AERs and ACRs are presented in Figs. 5 and 6, which demonstrate a heterogeneous but contextually meaningful pattern in the effectiveness of RA practices for improving SOC across diverse agro-ecological and agro-climatic conditions. The Eastern Plain (AER-13) and the corresponding Middle Gangetic Plain (ACR-4) emerged as high-response regions, with SOC effect sizes of 17.7 (k = 43, CI = $-2.22$ to 37.77) and 8.76 (k = 122, CI = $-3.38$ to 20.89), respectively. These regions are characterized by intensive rice–wheat systems where RA practices like residue incorporation, green manuring, and reduced tillage are combined. Such synergistic interventions have led to substantial gains in SOC, aligning with their high potential for RA scalability.

The Western Ghats and Coastal Plains (AER-19) and West Coast Plains & Ghat Region (ACR-12) also exhibited exceptional SOC improvements (estimate = 12.2 and 15.65, respectively). This can be attributed to favorable climatic conditions, high rainfall, and agroforestry legacies, which foster rapid organic matter turnover and microbial activity^58^. These areas highlight the natural affinity of humid ecosystems for carbon accumulation under regenerative land management. The semi-arid to sub-humid regions showed strong responses to RA practices as well. For instance, the Eastern Plateau (AER-11, estimate = 6.9, k = 57, CI = 0.1–13.71) and the Eastern Plateau & Hills (ACR-7, estimate = 6.18, k = 72, CI = 0.03–12.3) reported notable SOC increases, suggesting that degraded soils in these landscapes are highly responsive to organic inputs. Similarly, the Western Plateau & Hills (ACR-9, estimate = 8.12, k = 88, CI = 0.16–16.08), Upper Gangetic Plain (ACR-5, estimate = 6.71, k = 75, CI = 2.09–11.33), Trans-Gangetic Plain (ACR-6, estimate = 6.56, k = 181, CI = 0.10–13.02), and Gujarat Plains & Hills (ACR-13, estimate = 6.38, k = 28, CI = 1.74–11.01) demonstrated strong SOC benefits from RA.

Regions with moderate gains included the Deccan Plateau (AER-6 or ACR-9), Northern Plains and Central Highlands (AER-4 or ACR-5, 8), and Eastern Ghats & Tamil Nadu Uplands (AER-8, a part of ACR-10). Among these, semi-arid regions, often characterized by chronic drought and climatic unpredictability, stand to benefit from regenerative agriculture practices that enhance resilience; however, improvements in SOC are constrained by limited biomass inputs and irregular rainfall patterns. Notably, Assam and Bengal Plains (AER-15), characterized by high natural organic turnover, reported a modest effect (estimate = 4.43, k = 79, CI = 0.87–7.99), likely due to saturation effects in carbon accumulation. Similarly, the Western Himalayan region (AER-14, estimate = 3.98, k = 74, CI = 0.66–7.29) and Eastern/Western Himalayan regions (ACR-1 and ACR-2, $\sim$3.8) showed restrained SOC responses, reflecting altitudinal constraints, low decomposition rates, and limited biomass inputs in these cooler, high-elevation ecosystems.

## Discussion

This study represents one of the large-scale, India-specific meta-analyses systematically quantifying the impacts of multiple RA practices on SOC across diverse agro-ecological and climatic zones. By providing a comprehensive, regionally disaggregated evidence base, this work addresses a critical knowledge gap in the global literature, which has thus far been dominated by studies from temperate regions or mixed global datasets. The results highlight clear time-dependent trends in SOC sequestration, with rapid initial increases (within 5–-10 years) followed by a plateauing trend, consistent with carbon saturation theory and previous findings^11,59^. Early SOC gains were particularly pronounced in labile fractions under practices such as conservation tillage, residue retention, and cover cropping, while long-term accrual in more stable carbon pools was observed under integrated organic inputs and diversified rotations. These patterns indicate the importance of long-term monitoring to avoid overestimation of SOC persistence in short-term studies.

Among individual interventions, FYM application yielded the highest SOC gains, followed by green manure and compost. These differences reflect variations in organic matter quality, application rates, and decomposition dynamics. While compost contributes to stable carbon pools, its relatively lower field application rates may limit visible short-term effects. In contrast, FYM contains both labile and stable fractions, supporting microbial activity and contributing to SOC accumulation across fast- and slow-cycling pools^59^. Furthermore, synergistic combinations, such as FYM with cover crops or conservation tillage, were more effective than standalone practices, reaffirming the value of systems-level bundling for maximizing carbon outcomes^60^.

A key contribution of this study is the spatial disaggregation of SOC responses, revealing substantial heterogeneity across AERs and ACRs. Eastern India (e.g., AER-13, ACR-4) and the Western Ghats (AER-19, ACR-12) emerged as SOC “hotspots”, the areas where conditions such as high rainfall, dense vegetative cover, and favorable cropping systems (e.g., rice-wheat rotations, agroforestry) facilitate greater SOC accumulation and stabilization. Identifying such SOC hotspots is crucial for targeting nature-based climate solutions, as these regions offer higher carbon sequestration potential relative to others with less favorable biophysical conditions. In contrast, areas like the Central Highlands (AER-10) and the Telangana Plateau (AER-7) showed limited SOC gains, reflecting constraints such as moisture stress, shallow soils, and degraded land use history.

Residue retention proved particularly effective in high-productivity regions such as the Indo-Gangetic Plains and Eastern Plateau and Hills, where biomass returns are higher. In contrast, semi-arid regions like the Deccan Plateau and Western Dry Regions showed moderated responses due to water limitations that restrict biomass availability. In these cases, manure-based inputs like FYM and green manure were especially effective, compensating for minimal external nutrient inputs and widespread soil degradation^45,61^. Similarly, in humid and sub-humid zones such as the Eastern Coastal Plains and North-Eastern Hills, elevated microbial activity accelerates organic matter decomposition, necessitating sustained inputs and minimal disturbance to maintain SOC levels.

Importantly, these results demonstrate that no single RA practice is universally effective. Contextual tailoring is essential to maximize soil carbon gains. Humid and sub-humid areas, with high biomass potential, are ideal candidates for large-scale RA adoption and early inclusion in carbon markets. In contrast, semi-arid and dryland regions benefit more from integrated packages combining residue retention, water harvesting, and cover cropping to mitigate environmental limitations. Upland and plateau regions require adaptive strategies such as composting, agroforestry, and controlled grazing to boost biomass inputs and system resilience.

These findings carry significant implications for India’s national climate policy and sustainable agriculture programs. Practices such as FYM, green manure, conservation tillage, biochar, and integrated nutrient management have demonstrated consistent SOC benefits and could be considered for prioritization within government-supported RA initiatives. In alignment with the Government of India’s National Mission for Sustainable Agriculture (NMSA), which already promotes climate-resilient and resource-conserving farming practices, the wider adoption and scaling of RA interventions can further strengthen national efforts toward soil restoration, enhanced productivity, and sustainable agriculture. To accelerate adoption, particularly among smallholder farmers, policy tools need to consider offering targeted incentives. These could include equipment subsidies, community-scale biochar production, and composting training through self-help groups (SHGs).

Finally, it is important for climate finance mechanisms to consider the spatial heterogeneity in SOC sequestration potential. Region-specific evidence from this meta-analysis can help guide premium pricing for high-potential areas where regenerative practices consistently deliver strong SOC gains. At the same time, it can ensure more conservative crediting in marginal zones where biophysical constraints limit carbon sequestration. Without such differentiation, there is a risk of over-crediting in low-response regions, which could undermine the environmental integrity of carbon markets and lead to inflated claims of climate benefits. Precision-targeted investments can help balance these concerns while supporting the equitable and scalable adoption of regenerative farming that integrates sustainable land management with climate mitigation and resilience objectives.

## Conclusions

This meta-analysis, synthesizing evidence from 147 peer-reviewed studies across India’s diverse agro-ecological and agro-climatic regions, provides compelling support for RA as an effective strategy for enhancing SOC sequestration. Practices such as biochar application, FYM, green manure, conservation tillage, and fertilizer management consistently demonstrated significant SOC gains, with biochar, FYM, green manure, and fertilizer management ranking among the most effective. These results highlight the central role of organic inputs and reduced soil disturbance in restoring soil carbon and improving soil health.

The analysis also reveals that SOC accumulation is both time- and context-dependent. Greater gains were observed in long-term implementations ($>10$ years) and in specific regions, where soil, climate, and microbial dynamics favored carbon stabilization. While compost showed moderate effectiveness, its performance was influenced by application variability and the stabilized nature of its carbon pool. Crucially, the strong heterogeneity across regions underscores that blanket approaches to RA are sub-optimal. Instead, context-specific practice bundles, tailored to edaphic, climatic, and farming system conditions, are essential for maximizing carbon outcomes.

In conclusion, RA offers a scalable, nature-based solution for simultaneously addressing soil degradation, climate mitigation, and food system resilience. To unlock this potential, policies must prioritize high-impact practices, incentivize regionally adapted interventions, and invest in long-term research. Aligning RA implementation with national soil health and climate action goals could not only accelerate India’s progress toward carbon neutrality but also position it as a global exemplar of sustainable, carbon-smart agriculture.

## Methods

### Data sourcing and cleaning

A comprehensive and systematic literature search was conducted to identify peer-reviewed articles, reports, and conference proceedings that assessed the impact of RA practices on SOC. The search covered publications from year 2000 to 2024 and was performed using scientific databases including Scopus, Web of Science, and Google Scholar. Search terms included combinations of keywords such as: “regenerative agriculture”, “soil organic carbon”, “carbon sequestration”, “cover residue”, “zero tillage”, “organic farming”, “biochar”, “meta-analysis”, etc. Boolean operators and wildcards were used to ensure coverage (e.g., “carbon sequestration” AND “crop residue” OR “reduced tillage”). Duplicates were removed based on titles and abstracts, followed by full-text screening. Further, studies were selected for quantitative analysis based on predefined inclusion criteria: (1) reported measurements of soil carbon sequestration or SOC changes linked to regenerative practices; (2) provided quantitative data enabling calculation of effect sizes; (3) reported on field studies conducted in real-world agricultural settings; (4) covered at least one regenerative practice, such as cover cropping, no-till, crop rotation, or nutrient management; and (5) provided information on the duration of the study, location, and cropping system; Exclusion criteria included studies without sufficient quantitative data, greenhouse-based experiments, review papers, modeling-only studies, and those not reporting sufficient statistical data.

For each study, data were extracted on important variables, including study location, agro-ecological region, agro-climatic region, agricultural system (crop type, rotation patterns, etc.), RA practices applied, duration of study (number of years practices were implemented), etc. The experimental details such as the number of replications, mean values of SOC, standard deviations, and standard errors were collected to enable accurate estimation of effect sizes. To facilitate analysis and interpretation, RA practices were grouped into categories including: (1) tillage operations (minimun and zero), (2) manure application as organic amendments (FYM, compost, and green manure), (3) biochar application, (4) crop residue management, and (5) fertilizer management. Data extraction was performed by a reviewer, verified independently by another reviewer, and discrepancies were resolved through consensus or consultation. The number of replications was recorded for all studies where this information was available; however, studies lacking explicit replication details were retained to ensure broad coverage of the evidence base. In such cases, a standard assumption of three replications, common in agronomic field experiments, was applied for analytical consistency. In several of the studies included in the meta-analysis, data on standard deviations were not explicitly reported. For missing standard deviations, we imputed values by calculating the pooled standard deviation from similar studies within the same treatment group and agro-ecological region. When necessary, standard deviations were also estimated from reported confidence intervals or standard errors.

### Sensitivity analysis

To assess the robustness of the meta-analytic findings and identify studies exerting disproportionate influence on the overall effect size, a multi-step sensitivity analysis was conducted. The LOO approach was first employed, wherein each study was iteratively removed from the dataset to evaluate its impact on the pooled effect size. The average of all LOO estimates was considered as a benchmark to compare with the effect size estimate from each LOO iteration. The studies were flagged based on the magnitude of deviation from the benchmark: small deviation ($<0.05$), moderate deviation (0.05 to 0.1), and large deviation ($>0.1$).

The LOO results were complemented with the Baujat plot^62^ as a visual diagnostic tool to identify studies that contribute most to the overall heterogeneity (plotted on the x-axis) and have a substantial influence on the overall effect size (y-axis). The most influential studies, typically located in the upper-right quadrant of the plot, were flagged for further scrutiny. Finally, the influence() function from the metafor package in R^63^ was used to flag the influential data points. This method computes several statistics, including DFBETAS (Difference in Betas), Cook’s distance, and covariance ratios to assess the influence of individual studies on the model estimates. The internal ‘is.infl()’ function was used to flag studies that exceed established cut-off values based on these diagnostics. This multi-tiered sensitivity analysis provides a rigorous framework to detect and address the potential impact of influential studies, thereby strengthening the reliability of the meta-analytic conclusions.

### Meta-analysis

Meta-analyses were conducted using the metafor package in R^63^ employing a comprehensive suite of estimators to assess between-study variance ($\tau ^{2}$). Given the complexity of SOC response to RA practices across diverse contexts, multiple $\tau ^{2}$ estimators were compared to ensure robust and accurate synthesis of findings. The methods compared include: DerSimonian-Laird (DL): a commonly used method due to its simplicity and computational efficiency; Restricted Maximum Likelihood (REML): provides an unbiased estimate of $\tau ^{2}$ by accounting for the degrees of freedom used in estimating the fixed effects; Maximum Likelihood (ML): similar to REML but tends to underestimate $\tau ^{2}$ more in smaller samples; Sidik-Jonkman (SJ): designed to provide more accurate estimates when heterogeneity is present; Hedges (HE), an estimator that adjusts for small sample bias in the calculation of the standard error and is more conservative than DL in high-heterogeneity settings; Hunter-Schmidt (HS) and Hunter-Schmidt corrected (HSk): These are psychometric-based estimators, with HS focusing on correction for sampling error and HSk adding adjustments for measurement error, which is relevant when the precision of SOC measurements varies across studies; Empirical Bayes (EB): Offers a shrinkage-based estimation of $\tau ^{2}$, borrowing strength across studies to stabilize variance estimates, which is particularly beneficial in sparse or uneven datasets; Paule-Mandel (PM): A non-iterative estimator that performs well in both small and large samples and is considered a reliable alternative to DL or REML; and Paule-Mandel median-unbiased (PMM): it is a modified version of the PM estimator that aims to reduce bias, often used to derive more reliable confidence intervals and improve parameter estimation under uncertainty.

All models were implemented using the rma() function with the corresponding method specified. Estimators were compared based on $\tau ^{2}$ values, standard errors, and confidence interval widths. The model offering the best balance between bias, precision, and interpretability was selected for final analyses. Publication bias was assessed using funnel plots and Egger’s test. Additionally, a trim-and-fill method was applied to adjust for any detected bias, ensuring the reliability of the meta-analysis results^64^.

Following the identification of the most suitable approach based on comparative multimodel testing, a comprehensive subgroup analysis was done to unpack the contextual variability in the effectiveness of RA practices. These subgroupings were decided by study characteristics (e.g., study duration), intervention types (e.g., RA practices), and agro-ecological and climatic contexts relevant to Indian agriculture. This approach is particularly valuable in India, where farming systems are highly heterogeneous, and a majority of cultivators are smallholder and marginal farmers. Understanding the performance of RA practices under different scenarios is critical for targeted policy and program design.

**Table 3:** Summary statistics of meta-analysis by data point grouping. Values in parentheses represent analysis results after trim-fill bias correction.

Data points grouping	Estimate	SE	CI.lb	CI.ub	$\tau ^2$	$I^2$ (%)
All data points (k = 1020, filled = 296)	3.46 (1.98)	0.10 (0.15)	3.25 (1.68)	3.66 (2.29)	8.30 (27.2)	86.4 (94.2)
By studies (k = 146, filled = 47)	3.11 (1.98)	0.22 (0.31)	2.67 (1.38)	3.54 (2.59)	4.57 (13.8)	75.6 (88.4)
By study duration (k = 7, filled = 4)	5.21 (3.85)	1.09 (1.13)	3.07 (1.63)	7.35 (6.07)	2.32 (5.4)	29.5 (43.9)
By RA practices (k = 8, filled = 3)	4.21 (3.73)	0.78 (0.74)	2.68 (2.28)	5.75 (5.19)	0.00 (0.0)	0.0 (0.0)
By AER (k = 18, filled = 8)	3.43 (2.74)	0.46 (0.39)	2.54 (1.96)	4.32 (3.52)	0.54 (0.26)	15.4 (6.5)
By ACR (k = 13, filled = 6)	4.68 (3.96)	0.62 (0.56)	3.45 (2.85)	5.91 (5.07)	0.00 (0)	0.0 (0)
By study duration & RA practice (k = 32, filled = 13)	4.45 (3.25)	0.51 (0.51)	3.44 (2.25)	5.46 (4.27)	3.60 (5.4)	52.5 (57.3)
By AER & RA practice (k = 108, filled = 34)	3.31 (2.31)	0.27 (0.31)	2.77 (1.69)	3.84 (2.94)	4.81 (9.15)	73.5 (80.8)
By ACR & RA practice (k = 88, filled = 30)	3.69 (2.73)	0.29 (0.31)	3.11 (2.11)	4.28 (3.36)	4.04 (6.4)	64.6 (69.8)

## Data Availability

The datasets generated/extracted and/or analysed during the current study are available from the corresponding author on reasonable request.
